# Residential Neighborhood Disadvantage and Access to Kidney Transplantation

**DOI:** 10.1001/jamanetworkopen.2025.49679

**Published:** 2025-12-30

**Authors:** Yiting Li, Gayathri Menon, Byoungjun Kim, Sunjae Bae, Babak J. Orandi, Mario P. DeMarco, Wenbo Wu, Deidra C. Crews, Tanjala S. Purnell, Roland J. Thorpe, Sarah L. Szanton, Dorry L. Segev, Mara A. McAdams-DeMarco

**Affiliations:** 1 Department of Surgery, New York University Grossman School of Medicine, New York; 2 Department of Medicine, New York University Grossman School of Medicine, New York; 3 Department of Family Medicine and Community Health, University of Pennsylvania Perelman School of Medicine, Philadelphia; 4 Department of Population Health, New York University Grossman School of Medicine, New York; 5 Division of Nephrology, Johns Hopkins University School of Medicine, Baltimore, Maryland; 6 Division of Transplantation, Department of Surgery, Johns Hopkins University School of Medicine, Baltimore, Maryland; 7 Department of Epidemiology, Johns Hopkins Bloomberg School of Public Health, Baltimore, Maryland; 8 Department of Health Behavior and Society, Johns Hopkins Bloomberg School of Public Health, Baltimore, Maryland; 9 Johns Hopkins Alzheimer’s Disease Resource Center for Minority Aging Research, Johns Hopkins Bloomberg School of Public Health, Baltimore, Maryland; 10 Johns Hopkins Center for Health Disparities Solutions, Johns Hopkins Bloomberg School of Public Health, Baltimore, Maryland; 11 Johns Hopkins University School of Nursing, Baltimore, Maryland

## Abstract

**Importance:**

Residence in a disadvantaged neighborhood is a key driver of racial and ethnic disparities in the diagnosis and management of chronic diseases; however, its impact on disparities in access to waitlisting and kidney transplantation (KT) is unclear.

**Objective:**

To examine the association between neighborhood disadvantage and access to waitlisting and KT.

**Design, Setting, and Participants:**

This retrospective cohort study (January 1, 2015, to December 31, 2021) used a US national registry to assess adults (aged ≥18 years) with end-stage kidney disease (ESKD) and adult KT candidates. Statistical analysis was performed in March 2025.

**Exposure:**

Residential neighborhood disadvantage score (built environment disadvantage, criminal injustice, education disadvantage, unemployment, housing instability, poverty, social fragmentation, transportation barrier, and wealth inequality) ascertained by American Community Survey and other public data sources.

**Main Outcomes and Measures:**

The adjusted hazard ratios (AHRs) of waitlisting and KT (any KT, live-donor KT [LDKT], and preemptive KT) were assessed across tertiles of the neighborhood disadvantage score using cause-specific hazard models. Interaction terms were used to quantify these aforementioned associations by race and ethnicity.

**Results:**

The study included 501 444 adults with ESKD initiating dialysis (mean [SD] age, 63.9 [14.6] years; 293 937 [58.6%] male; 25 790 [5.1%] Asian [Asian American, Native Hawaiian, and Pacific Islander], 133 923 [26.7%] Black, 66 323 [13.2%] Hispanic, and 275 408 [54.9%] White) and 95 068 KT candidates on the waitlist (mean [SD] age, 53.7 [13.0] years; 60 328 [63.5%] male; 6956 [7.3%] Asian, 25 215 [26.5%] Black, 15 685 [16.5%] Hispanic, and 47 212 [49.7%] White). A total of 173 880 adults with ESKD (34.7%) and 26 718 KT candidates (28.1%) resided in high-disadvantage neighborhoods. After adjustment, adults residing in high-disadvantage neighborhoods were less likely to be waitlisted (AHR, 0.71; 95% CI, 0.69-0.72) compared with those in low-disadvantage neighborhoods. Specifically, Asian (AHR, 0.87; 95% CI, 0.80-0.95), Black (AHR, 0.68; 95% CI, 0.66-0.70), Hispanic (AHR, 0.89; 95% CI, 0.86-0.92), and White (AHR, 0.68; 95% CI, 0.66-0.71) adults in high-disadvantage neighborhoods were less likely to be waitlisted compared with White adults in low-disadvantage neighborhoods. Overall, candidates residing in high-disadvantage neighborhoods were less likely to receive any KT (AHR, 0.89; 95% CI, 0.87-0.92), LDKT (AHR, 0.65; 95% CI, 0.62-0.69), and preemptive KT (AHR, 0.62; 95% CI, 0.58-0.67). Notably, Black candidates residing in high-disadvantage neighborhoods were less likely to receive KT (AHR, 0.60; 95% CI, 0.58-0.62), LDKT (AHR, 0.23; 95% CI, 0.21-0.25), and preemptive KT (AHR, 0.22; 95% CI, 0.20-0.25) compared with White candidates in low-disadvantage neighborhoods.

**Conclusions and Relevance:**

In this cohort study of adults with ESKD and KT candidates, residence in high-disadvantage neighborhoods was associated with reduced access to waitlisting and KT; it also was associated with persistent racial and ethnic disparities in LDKT and preemptive KT. These results suggest that to support equitable access, clinicians and transplant programs should work with social workers and community advocates to implement initiatives (eg, outreach and financial support) that address structural barriers and direct resources to affected neighborhoods.

## Key Points

**Question:**

Does residing in a high-disadvantage neighborhood affect access to waitlisting and kidney transplantation (KT)?

**Findings:**

This cohort study found that for adults with end-stage kidney disease, residence in high-disadvantage neighborhoods was associated with a significantly lower likelihood of waitlisting, any KT, live-donor KT, and preemptive KT. The associations between neighborhood disadvantage and KT access were most pronounced among minoritized patients and those residing in the Western regions of the US, and residence in suburban or rural high-disadvantage neighborhoods was associated with especially low access to live-donor KT.

**Meaning:**

The findings suggest that residence in high-disadvantage neighborhoods is associated with reduced access to waitlisting and KT and contributes to persistent racial and ethnic disparities in access to live-donor KT and preemptive KT.

## Teaser

This cohort study of adults with end-stage kidney disease or kidney transplantation candidates assessed the association between neighborhood disadvantage and access to waitlisting and kidney transplantation.

## Introduction

Kidney transplantation (KT) is the preferred treatment option for individuals with end-stage kidney disease (ESKD), conferring better life expectancy and quality of life than dialysis.^1,2,3,4,5^ Live donor KT (LDKT) and preemptive KT are the ideal forms of transplantation: LDKT offers shorter waitlist times and improved patient and graft survival,^1,2,3,4,6,7^ whereas preemptive KT avoids dialysis-related risk, resulting in fewer complications and lower costs.^8,9,10^ However, despite extensive efforts to increase equity in transplantation,^1,2,3,11^ including the 2014 Kidney Allocation System to reduce waiting times and other interventions,^1,2,3,11^ substantial racial and ethnic disparities in KT remain. Black and Hispanic adults with ESKD continue to have lower access to waitlist, LDKT, and preemptive KT compared with White adults,^12^ suggesting that broader systemic factors may be at play.

Although the harmful effects of structural barriers (eg, neighborhood disadvantage) on health disparities are well documented,^13,14^ their impact on disparities in access to KT remains underexplored. Disadvantaged neighborhoods, shaped by systematic and institutionalized discrimination experienced by certain racial or ethnic groups,^6,13,15,16,17^ perpetuate disparities through interdependent inequitable systems, such as housing, employment, education, income, and criminal justice.^6,15,17^ These systems then reinforce discriminatory beliefs and unequal resource distribution, ultimately increasing the risk of adverse health outcomes.^6,13,15,17^ Particularly, minoritized individuals residing in disadvantaged neighborhoods often face greater socioeconomic deprivation,^8,13,17,18^ inadequate housing,^18^ reduced health literacy,^8,18^ limited access to quality health care,^8,18^ and delayed disease diagnosis,^8,13^ contributing to a higher comorbidity burden.^13,18,19^ Together, these barriers may directly influence patients’ eligibility for waitlisting and transplantation (eg, financial instability or difficulty adhering to medical care)^8,17,20,21^ and may vary by regions and urbanicity. In this national study, we examined the association between neighborhood disadvantage and access to waitlisting and KT. We also examined these associations by race and ethnicity, US region, and urbanicity.

## Methods

### Study Population and Data Source

We leveraged data from the United States Renal Data System (USRDS) to study adults (aged ≥18 years) with incidentally diagnosed ESKD in the US who initiated dialysis between January 1, 2015, and December 31, 2021. This study consisted of 2 analytical cohorts: the dialysis cohort (501 444 adults with ESKD initiating dialysis to quantify access to waitlisting) and the waitlisted cohort (95 068 adults waitlisted for a KT [candidates] to quantify access to transplantation). The study population was limited to adults whose race and ethnicity were categorized into one of the following categories (practitioner-reported race and ethnicity information from USRDS patient files): non-Hispanic Asian (Asian hereafter), non-Hispanic Black (Black hereafter), Hispanic, or non-Hispanic White (White hereafter). The USRDS is a national registry that contains comprehensive information on all adults with ESKD in the US, including details from Medicare claims.^22^ We ascertained patient characteristics and comorbidities from the Centers for Medicare & Medicaid Services medical evidence form at dialysis initiation,^22^ patient files, and waitlist and transplant characteristics from the United Network for Organ Sharing and Organ Procurement and Transplantation Network records.^22^

Our study was reviewed by the New York University Grossman School of Medicine institutional review board and considered exempt because the patients could not be identified, thus waiving the need for informed consent. We followed the Strengthening the Reporting of Observational Studies in Epidemiology (STROBE) reporting guideline for cohort studies. The clinical and research activities being reported are consistent with the Declaration of Istanbul as outlined in the Declaration of Istanbul on Organ Trafficking and Transplant Tourism.^23^

### Neighborhood Disadvantage Score 

The neighborhood disadvantage score, a previously published and validated measure, was determined using the American Community Survey and other public data sources.^15,24^ Nine standardized domains were identified a priori through a literature review on structural racism and social determinants of health^15^: built environment disadvantage, criminal injustice, education disadvantage, unemployment, housing instability, poverty, social fragmentation, transportation barrier, and wealth inequality. This was explicitly constructed using a structural racism framework to create a multidimensional summary measure, addressing limitations of existing indexes (eg, Area Deprivation Index and Social Vulnerability Index) (eTable 1 in Supplement 1).^15^ Both domain- and summary-level scores were then aggregated to the Zip Code Tabulation Area (ZCTA) level by taking population-weighted means of the intersecting census tracts.^15^ For our study, these precalculated scores were subsequently linked to individuals’ zip codes using a zip-to-ZCTA crosswalk.^15,24^

We assigned the neighborhood disadvantage scores to adults with ESKD based on their 5-digit zip code at the time of dialysis initiation (dialysis cohort) and to adults waitlisted for KT based on their zip code at the time of waitlisting (waitlisted cohort). Consistent with prior studies,^25,26,27,28,29,30^ we split neighborhood disadvantage scores into tertiles: low (≤−0.116), medium (>−0.116 and ≤0.681), and high (>0.681).

### Patient Characteristics

From the existing literature on neighborhood disadvantage in KT access,^6,16^ we selected the following covariates: age, sex, body mass index, cause of ESKD, the year of dialysis initiation or waitlisting, employment status, pre-ESKD nephrologic care, comorbidities, and functional status. Blood group and calculated panel reactive antibody were used only for the waitlisted cohort to quantify access to KT. Neighborhood racial and ethnic composition was not included as a covariate due to multicollinearity with the social fragmentation domain (residential segregation) of neighborhood disadvantage score.

### Access to Waitlisting and KT

The outcomes were first waitlisting after dialysis initiation (dialysis cohort) and receipt of first KT (any KT, LDKT, and preemptive KT) after waitlisting (waitlisted cohort). Preemptive KT is defined as receiving a transplant without prior dialysis, based on USRDS reporting.^12^ All outcomes were ascertained using dialysis, waitlisting, and transplantation dates from the national registry.

### Statistical Analysis

We used the Kaplan-Meier method to estimate the 5-year unadjusted cumulative incidence of waitlisting after dialysis initiation and of first KT after waitlisting. Cause-specific hazards models were used to quantify the following: (1) the association between neighborhood disadvantage score tertiles and time to first waitlisting and KT; (2) the difference in these associations by race and ethnicity, US Census regions, and urbanicity (modified Rural-Urban Commuting Area Codes: high-density urban, suburban, rural, and small town),^31,32^ using interaction terms and Wald tests; and (3) the individual domains of the neighborhood disadvantage score to identify those most salient to the outcomes. We used complementary log-log plots and Schoenfeld residuals to test the proportional hazards assumption.

For the outcome of waitlisting, we followed up adults from their date of dialysis initiation to the date of their first waitlisting, censoring at the earliest occurrence of the following: KT without listing, death, or end of follow-up (December 31, 2021). For the outcome of KT, we followed up candidates on the waitlist from their first waitlisting date to the date of their first KT, censoring at the earliest occurrence of the following: waitlist removal, death, or end of follow-up (December 31, 2021). For the outcome of LDKT, we followed up candidates on the waitlist from their first waitlisting date to the date of their first LDKT, censoring at the earliest occurrence of the following: waitlist removal, deceased donor KT, preemptive KT, death, or end of follow-up. Candidates who received a preemptive KT were followed up from their first waitlisting date to the date of their first preemptive KT, censoring at the earliest occurrence of the following: waitlist removal, deceased donor KT, LDKT, death, or end of follow-up. Individuals who received LDKT and preemptive KT without being waitlisted earlier were assigned 1 day at risk.

We assessed the robustness of our findings through the following sensitivity analyses: using Fine and Gray proportional subdistribution hazards models (competing risk of death) and splitting neighborhood disadvantage score into tertiles based on the national mean: low (≤−0.2), medium (>−0.2 and ≤0.52), and high (>0.52).^15^ All statistical analyses were conducted using SAS software, version 9.4 (SAS Institute Inc) and Stata software, release 17 (StataCorp). Statistical significance was defined as a 2-sided *P* < .05.

## Results

### Dialysis Cohort

The study included 501 444 adults with ESKD initiating dialysis (mean [SD] age, 63.9 [14.6] years; 293 937 [58.6%] male and 207 507 [41.4%]; 25 790 [5.1%] Asian [Asian American, Native Hawaiian, and Pacific Islander], 133 923 [26.7%] Black, 66 323 [13.2%] Hispanic, and 275 408 [54.9%] White). A total of 173 880 adults with ESKD (34.7%) resided in high-disadvantage neighborhoods. Additionally, 241 720 (48.2%) had diabetes, 154 775 (30.9%) had hypertension as the cause of ESKD, and 329 599 (65.7%) had pre-ESKD nephrology care (Table 1).

**Table 1.** Characteristics of Adults With ESKD Initiating Dialysis Between 2015 and 2021, Stratified by Residential Neighborhood Disadvantage Score

Characteristic	No. (%) of patients			
	Patients with ESKD (n = 501 444)	Neighborhood disadvantage score^a^		
		Low (n = 162 781)	Medium (n = 164 783)	High (n = 173 880)
Age, mean (SD), y	63.9 (14.6)	61.3 (14.4)	66.9 (14.3)	63.6 (14.4)
Age group, y				
18-34	20 361 (4.1)	5091 (3.1)	6790 (4.1)	8480 (4.9)
35-49	61 957 (12.4)	14 563 (8.9)	20 404 (12.4)	26 990 (15.5)
50-64	156 314 (31.2)	42 672 (26.2)	52 259 (31.7)	61 383 (35.3)
≥65	262 812 (52.4)	100 455 (61.7)	85 330 (51.8)	77 027 (44.3)
Sex				
Male	293 937 (58.6)	99 507 (61.1)	96 851 (58.8)	97 579 (56.1)
Female	207 507 (41.4)	63 274 (38.9)	67 932 (41.2)	76 301 (43.9)
Race and ethnicity				
Asian^b^	25 790 (5.1)	15 052 (9.2)	7540 (4.6)	3198 (1.8)
Black	133 923 (26.7)	25 681 (15.8)	36 283 (22.0)	71 959 (41.4)
Hispanic	66 323 (13.2)	14 382 (8.8)	22 727 (13.8)	29 214 (16.8)
White	275 408 (54.9)	107 666 (66.1)	98 233 (59.6)	69 509 (40.0)
BMI, mean (SD)	29.9 (8.1)	30.4 (8.3)	29.2 (7.7)	30.2 (8.1)
BMI group				
≤25	150 169 (29.9)	53 440 (32.8)	47 577 (28.9)	49 152 (28.3)
26-30	139 375 (27.8)	46 604 (28.6)	45 329 (27.5)	47 442 (27.3)
>30	211 900 (42.3)	62 737 (38.5)	71 877 (43.6)	77 286 (44.4)
Employment status				
Unemployed	114 526 (22.8)	27 591 (16.9)	36 324 (22.0)	50 611 (29.1)
Employed	57 457 (11.5)	20 611 (12.7)	19 205 (11.7)	17 641 (10.1)
Retired	306 491 (61.1)	107 363 (66.0)	101 421 (61.5)	97 707 (56.2)
Other	22 970 (4.6)	7216 (4.4)	7833 (4.8)	7921 (4.6)
Cause of ESKD				
Diabetes	241 720 (48.2)	74 084 (45.5)	81 223 (49.3)	86 413 (49.7)
Hypertension	154 775 (30.9)	47 812 (29.4)	48 425 (29.4)	58 538 (33.7)
Glomerulonephritis	31 723 (6.3)	12 005 (7.4)	10 658 (6.5)	9060 (5.2)
Other	73 226 (14.6)	28 880 (17.7)	24 477 (14.9)	19 869 (11.4)
Comorbidities				
Cancer	36 375 (7.3)	14 519 (8.9)	11 932 (7.2)	9924 (5.7)
Peripheral vascular disease	46 653 (9.3)	15 174 (9.3)	15 975 (9.7)	15 504 (8.9)
Cerebrovascular disease	44 873 (8.9)	13 615 (8.4)	14 849 (9.0)	16 409 (9.4)
Atherosclerotic heart disease	65 602 (13.1)	23 943 (14.7)	21 749 (13.2)	19 910 (11.5)
CHF	147 717 (29.5)	47 967 (29.5)	48 619 (29.5)	51 131 (29.4)
COPD	47 594 (9.5)	13 979 (8.6)	16 889 (10.2)	16 726 (9.6)
Drug use	6151 (1.2)	1365 (0.8)	1943 (1.2)	2843 (1.6)
Alcohol use	7765 (1.5)	2324 (1.4)	2534 (1.5)	2907 (1.7)
Tobacco use	33815 (6.7)	7779 (4.8)	11 895 (7.2)	14 141 (8.1)
Functional impairment	78 585 (15.7)	24 537 (15.1)	26 363 (16.0)	27 685 (15.9)
Institutionalized	40 875 (8.2)	14 055 (8.6)	13 953 (8.5)	12 867 (7.4)
Pre-ESKD nephrologic care	329 599 (65.7)	112 570 (69.2)	109 930 (66.7)	107 099 (61.6)
Urbanicity^c^				
High-density urban	190 070 (37.9)	73 111 (44.9)	58 186 (35.3)	58 773 (33.8)
Suburban	173 954 (34.7)	67 389 (41.4)	57 831 (35.1)	48 734 (28.0)
Rural	75 188 (15.0)	16 271 (10.0)	26 324 (16.0)	32 593 (18.7)
Small town	62 232 (12.4)	6010 (3.7)	22 442 (13.6)	33 780 (19.4)

Abbreviations: BMI, body mass index (calculated as weight in kilograms divided by height in meters squared); CHF, congestive heart failure; COPD, chronic obstructive pulmonary disease; ESKD, end-stage kidney disease.

^a^ Neighborhood disadvantage score: 9 domains (built environment disadvantage, criminal injustice, education disadvantage, unemployment, housing instability, poverty, social fragmentation, transportation barrier, and wealth inequality). Because increasing values of the 9 domains indicated more disadvantages, the naming convention was revised to reflect the appropriate interpretation of the domains and to improve clarity.^15^

^b^ Asian: Asian American, Native Hawaiian, and Pacific Islander.

^c^ This classification modified the original 2010 Rural-Urban Commuting Area Codes defined by US Department of Agriculture.^31^

### Access to Waitlisting

The 5-year unadjusted cumulative incidence of waitlisting was lowest for adults residing in high-disadvantage neighborhoods (high, 20.3% [95% CI, 20.1%-20.7%]; low, 23.5% [95% CI, 23.2%-23.9%]; log-rank *P* < .001) (Figure). After adjustment, adults with ESKD residing in high-disadvantage neighborhoods had a lower likelihood of waitlisting (adjusted hazard ratio [AHR], 0.71; 95% CI, 0.69-0.72), and this association differed by race and ethnicity (*P* < .001 for interaction) (Table 2). Within each racial and ethnic group, Asian (AHR, 0.76; 95% CI, 0.70-0.84), Black (AHR, 0.69; 95% CI, 0.67-0.72), Hispanic (AHR, 0.79; 95% CI, 0.76-0.83), and White (AHR, 0.68; 95% CI, 0.66-0.71) adults in high-disadvantage neighborhoods had a lower likelihood of waitlisting compared with their counterparts in low-disadvantage neighborhoods (Table 2). In cross–race and ethnicity comparisons, compared with White adults in low-disadvantage neighborhoods, Asian (AHR, 0.87; 95% CI, 0.80-0.95), Black (AHR, 0.68; 95% CI, 0.66-0.70), and Hispanic (AHR, 0.89; 95% CI, 0.86-0.92) adults in high-disadvantage neighborhoods had a lower likelihood of waitlisting (*P* < .001 for interaction) (Table 2). Adults with ESKD residing in high-disadvantage neighborhoods in the Midwest (AHR, 0.57; 95% CI, 0.55-0.60), South (AHR, 0.60; 95% CI, 0.58-0.62), or West (AHR, 0.57; 95% CI, 0.55-0.60) regions of the US had a lower likelihood of waitlisting compared with those in low-disadvantage neighborhoods in the Northeast (*P* = .01 for interaction) (eTable 2 in Supplement 1). Furthermore, the association of neighborhood disadvantage with waitlisting did not differ by urbanicity (*P* = .59 for interaction) (eTable 2 in Supplement 1). All domains of neighborhood disadvantage were associated with access to waitlisting, with the strongest associations observed for increasing disadvantage in built environment (AHR, 0.86; 95% CI, 0.85-0.87), education disadvantage (AHR, 0.85; 95% CI, 0.84-0.86), unemployment (AHR, 0.87; 95% CI, 0.86-0.88), housing instability (AHR, 0.85; 95% CI, 0.84-0.86), and wealth inequality (AHR, 0.83; 95% CI, 0.82-0.84) (Table 3).

**Figure.**
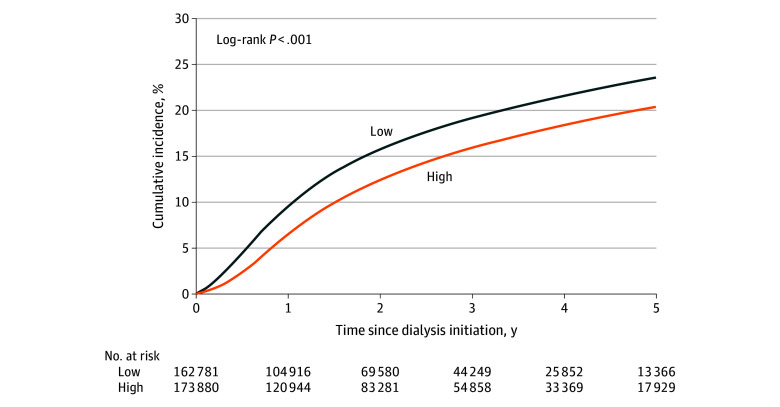
Residential Neighborhood Disadvantage Score and Time to Listing Among Adults With End-Stage Kidney Disease Initiating Dialysis (2015-2021) For ease of interpretation, we excluded the medium neighborhood disadvantage score from the figure.

**Table 2.** Residential Neighborhood Disadvantage and Time to Listing Among Adults With End-Stage Kidney Disease Initiating Dialysis, Stratified by Race and Ethnicity, US Region, and Urbanicity (2015-2021)^**a**^

Listed (n = 501 444)	Residential neighborhood disadvantage score, AHR (95% CI)^b^		
	Low	Medium	High
Overall	1.0 [Reference]	0.83 (0.81-0.84)^c^	0.71 (0.69-0.72)^c^
Race and ethnicity			
Asian^d^	1.14 (1.10-1.19)^c^	1.00 (0.95-1.06)^c^	0.87 (0.80-0.95)^c^
Black	0.98 (0.95-1.02)^c^	0.84 (0.82-0.87)^c^	0.68 (0.66-0.70)^c^
Hispanic	1.12 (1.08-1.17)^c^	0.96 (0.92-0.99)^c^	0.89 (0.86-0.92)^c^
White	1.0 [Reference]	0.80 (0.78-0.82)^c^	0.68 (0.66-0.71)^c^
*P* value for interaction^e^	<.001^c^	<.001^c^	<.001^c^
Asian^d^	1.0 [Reference]	0.87 (0.82-0.93)^c^	0.76 (0.70-0.84)^c^
Black	1.0 [Reference]	0.86 (0.83-0.89)^c^	0.69 (0.67-0.72)^c^
Hispanic	1.0 [Reference]	0.85 (0.82-0.89)^c^	0.79 (0.76-0.83)^c^
White	1.0 [Reference]	0.80 (0.78-0.82)^c^	0.68 (0.66-0.71)^c^
*P* value for interaction^e^	NA	<.001^c^	<.001^c^

Abbreviations: AHR, adjusted hazard ratio; NA, not applicable.

^a^ Cause-specific hazards models were adjusted for year of dialysis initiation, age, sex, cause of end-stage kidney disease, employment status, body mass index, nephrology care, comorbidities (cancer, hypertension, diabetes, peripheral vascular disease, atherosclerotic heart disease, congestive heart failure, chronic obstructive pulmonary disease, drug use, alcohol use, and tobacco use), and functional status.

^b^ Neighborhood disadvantage score: 9 domains (built environment disadvantage, criminal injustice, education disadvantage, unemployment, housing instability, poverty, social fragmentation, transportation barrier, and wealth inequality). Because increasing values of the 9 domains indicated more disadvantages, the naming convention was revised to reflect the appropriate interpretation of the domains and to improve clarity.^15^

^c^ Associations that are statistically significant (*P* < .05).

^d^ Asian: Asian American, Native Hawaiian, and Pacific Islander.

^e^ *P* value for the interaction between neighborhood disadvantage and race and ethnicity.

**Table 3.** Components of Residential Neighborhood Disadvantage Score and Time to Listing, LDKT, and Preemptive KT Among Adults With End-Stage Kidney Disease Initiating Dialysis or Adult KT Candidates (2015-2021)

Component	AHR (95% Cl)		
	Listing (n = 501 444)^a^	LDKT (n = 95 068)^b^	Preemptive KT (n = 95 068)^b^
Neighborhood disadvantage score domain^c^			
Built environment disadvantage	0.86 (0.85-0.87)^d^	0.84 (0.82-0.86)^d^	0.82 (0.79-0.84)^d^
Criminal injustice	0.96 (0.95-0.97)^d^	0.99 (0.97-1.00)^d^	0.94 (0.92-0.96)^d^
Education disadvantage	0.85 (0.84-0.86)^d^	0.83 (0.81-0.85)^d^	0.78 (0.77-0.80)^d^
Unemployment	0.87 (0.86-0.88)^d^	0.86 (0.84-0.87)^d^	0.83 (0.82-0.85)^d^
Housing instability	0.85 (0.84-0.86)^d^	0.79 (0.77-0.81)^d^	0.79 (0.76-0.81)^d^
Poverty	0.84 (0.83-0.85)^d^	0.79 (0.78-0.81)^d^	0.75 (0.73-0.78)^d^
Social fragmentation	0.88 (0.87-0.89)^d^	0.81 (0.79-0.83)^d^	0.78 (0.76-0.81)^d^
Transportation barrier	0.91 (0.90-0.92)^d^	0.92 (0.90-0.94)^d^	0.90 (0.88-0.93)^d^
Wealth inequality	0.83 (0.82-0.84)^d^	0.88 (0.86-0.89)^d^	0.86 (0.84-0.88)^d^

Abbreviations: AHR, adjusted hazard ratio; KT, kidney transplant; LDKT, live-donor kidney transplant.

^a^ Cause-specific hazards models adjusted for year of dialysis initiation, age, sex, cause of end-stage kidney disease, employment status, body mass index, nephrologic care, comorbidities (cancer, hypertension, diabetes, peripheral vascular disease, atherosclerotic heart disease, congestive heart failure, chronic obstructive pulmonary disease, drug use, alcohol use, and tobacco use), and functional status.

^b^ Cause-specific hazards models adjusted for year of listing, age at listing, sex, cause of end-stage kidney disease, body mass index, blood type, peak reactivity antibody, comorbidities (cancer, hypertension, diabetes, peripheral vascular disease, atherosclerotic heart disease, congestive heart failure, chronic obstructive pulmonary disease, drug use, alcohol use, and tobacco use), and functional status.

^c^ Neighborhood disadvantage score: 9 domains (built environment disadvantage, criminal injustice, education disadvantage, unemployment, housing instability, poverty, social fragmentation, transportation barrier, and wealth inequality). Because increasing values of the 9 domains indicated more disadvantages, the naming convention was revised to reflect the appropriate interpretation of the domains and to improve clarity. Each domain’s measures were standardized.^15^

^d^ Associations that are statistically significant (*P* < .05).

### Waitlisted Cohort

Among 95 068 KT candidates on the waitlist, the mean (SD) age was 53.7 (13.0) years, 60 328 (63.5%) were male and 34 740 (36.5%) female, 6956 (7.3%) were Asian, 25 215 (26.5%) were Black, 15 685 (16.5%) were Hispanic, and 47 212 (49.7%) were White. Additionally, 35 137 (37.0%) had diabetes and 25 701 (27.0%) had hypertension as the cause of ESKD (eTable 3 in Supplement 1).

### Access to KT

#### Any KT

The 5-year unadjusted cumulative incidence of any KT (high, 62.5% [95% CI, 61.5%-63.6%]; low, 70.1% [95% CI, 69.9%-71.5%]; log-rank *P* < .001) was lowest for candidates residing in high-disadvantage neighborhoods. After adjustment, residence in high-disadvantage neighborhoods was associated with a lower likelihood of any KT (AHR, 0.89; 95% CI, 0.87-0.92). In cross–race and ethnicity comparisons, compared with White candidates in low-disadvantage neighborhoods, Asian (AHR, 0.57; 95% CI, 0.51-0.64), Black (AHR, 0.60; 95% CI, 0.58-0.62), and Hispanic (AHR, 0.62; 95% CI, 0.59-0.65) candidates residing in high-disadvantage neighborhoods had a lower likelihood of any KT (*P* = .04 for interaction) (eTable 4 in Supplement 1). KT candidates residing in high-disadvantage neighborhoods in the West region of the US had a lower likelihood of any KT (AHR, 0.67; 95% CI, 0.63-0.72; *P* < .001 for interaction) compared with those in low-disadvantage neighborhoods in the Northeast. However, the impact of neighborhood disadvantage on any KT did not differ by urbanicity (*P* = .69 for interaction) (eTable 4 in Supplement 1). The strongest associations were observed for increasing education disadvantage (AHR, 0.94; 95% CI, 0.93-0.95), housing instability (AHR, 0.91; 95% CI, 0.90-0.93), and poverty (AHR, 0.92; 95% CI, 0.91-0.93) (eTable 5 in Supplement 1).

#### LDKT and Preemptive KT

The 5-year unadjusted cumulative incidence of LDKT (high, 14.3% [95% CI, 13.6%-15.1%]; low, 26.9% [95% CI, 26.2%-27.8%]; log-rank *P* < .001) (eFigure 1A in Supplement 1) and preemptive KT (high, 6.8% [95% CI, 6.3%-7.3%]; low, 18.3% [95% CI, 17.7%-18.9%]; log-rank *P* < .001) (eFigure 1B in Supplement 1) were lowest for candidates residing in high-disadvantage neighborhoods. After adjustment, candidates residing in high-disadvantage neighborhoods had a lower likelihood of LDKT (AHR, 0.65; 95% CI, 0.62-0.69) and preemptive KT (AHR, 0.62; 95% CI, 0.58-0.67) (Table 4). Specifically, compared with White candidates in low-disadvantage neighborhoods, Asian, Black, and Hispanic candidates residing in high-disadvantage neighborhoods had a lower likelihood of LDKT (Asian: AHR, 0.25; 95% CI, 0.18-0.34; Black: AHR, 0.23; 95% CI, 0.21-0.25; Hispanic: AHR, 0.51; 95% CI, 0.47-0.56; *P* < .001 for interaction), and preemptive KT (Asian: AHR, 0.20; 95% CI, 0.13-0.32; Black: AHR, 0.22; 95% CI, 0.20-0.25; Hispanic: AHR, 0.25; 95% CI, 0.21-0.29; *P* = .002 for interaction) (Table 4). Candidates residing in high-disadvantage neighborhoods in the West region of the US had a lower likelihood of LDKT (AHR, 0.43; 95% CI, 0.38-0.49; *P* < .001 for interaction), and preemptive KT (AHR, 0.45; 95% CI, 0.38-0.54; *P *< .001 for interaction) compared with those in low-disadvantage neighborhoods in the Northeast (eTable 2 in Supplement 1). Furthermore, the impact of neighborhood disadvantage on LDKT differed by urbanicity (*P* < .001 for interaction), whereas for preemptive KT it did not (*P* = .59 for interaction) (eTable 2 in Supplement 1). All domains of neighborhood disadvantage were associated with access to LDKT and preemptive KT (Table 3).

**Table 4.** Residential Neighborhood Disadvantage and Time to LDKT and Preemptive KT Among KT Candidates, Stratified by Race and Ethnicity, US Region, and Urbanicity (2015-2021)^**a**^

Characteristic	Residential neighborhood disadvantage score, AHR (95% CI)^b^					
	LDKT			Preemptive KT		
	Low	Medium	High	Low	Medium	High
Overall	1.0 [Reference]	0.82 (0.78-0.85)^c^	0.65 (0.62-0.69)^c^	1.0 [Reference]	0.75 (0.72-0.79)^c^	0.62 (0.58-0.67)^c^
Race and ethnicity						
Asian^d^	0.44 (0.40-0.48)^c^	0.37 (0.31-0.43)^c^	0.25 (0.18-0.34)^c^	0.39 (0.34-0.44)^c^	0.31 (0.24-0.39)^c^	0.20 (0.13-0.32)^c^
Black	0.48 (0.44-0.53)^c^	0.32 (0.29-0.35)^c^	0.23 (0.21-0.25)^c^	0.29 (0.26-0.33)^c^	0.25 (0.22-0.29)^c^	0.22 (0.20-0.25)^c^
Hispanic	0.69 (0.63-0.75)^c^	0.55 (0.50-0.60)^c^	0.51 (0.47-0.56)^c^	0.51 (0.45-0.57)^c^	0.35 (0.31-0.40)^c^	0.25 (0.21-0.29)^c^
White	1.0 [Reference]	0.85 (0.80-0.89)^c^	0.69 (0.64-0.73)^c^	1.0 [Reference]	0.75 (0.71-0.79)^c^	0.63 (0.58-0.68)^c^
*P* value for interaction^e^	<.001^c^	<.001^c^	<.001^c^	<.001^c^	<.001^c^	.002^c^
Asian^d^	1.0 [Reference]	1.12 (1.07-1.17)^c^	0.97 (0.91-1.03)	1.0 [Reference]	1.07 (1.01-1.13)^c^	0.98 (0.91-1.06)
Black	1.0 [Reference]	0.52 (0.48-0.57)^c^	0.48 (0.45-0.52)^c^	1.0 [Reference]	0.56 (0.51-0.61)^c^	0.51 (0.46-0.56)^c^
Hispanic	1.0 [Reference]	0.73 (0.68-0.79)^c^	0.71 (0.66-0.77)^c^	1.0 [Reference]	0.64 (0.58-0.71)^c^	0.61 (0.55-0.67)^c^
White	1.0 [Reference]	0.85 (0.80-0.89)^c^	0.69 (0.64-0.73)^c^	1.0 [Reference]	0.75 (0.71-0.79)^c^	0.63 (0.58-0.68)^c^
*P* value for interaction^e^	NA	<.001^c^	.001^c^	NA	<.001^c^	<.001^c^

Abbreviations: AHR, adjusted hazard ratio; NA, not applicable.

^a^ Cause-specific hazards models adjusted for year of listing, age at listing, sex, cause of end-stage kidney disease, body mass index, blood type, peak reactivity antibody, comorbidities (cancer, hypertension, diabetes, peripheral vascular disease, atherosclerotic heart disease, congestive heart failure, chronic obstructive pulmonary disease, drug use, alcohol use, and tobacco use), and functional status.

^b^ Neighborhood disadvantage score: 9 domains (built environment disadvantage, criminal injustice, education disadvantage, unemployment, housing instability, poverty, social fragmentation, transportation barrier, and wealth inequality). Because increasing values of the 9 domains indicated more disadvantages, the naming convention was revised to reflect the appropriate interpretation of the domains and to improve clarity.^15^

^c^ Associations that are statistically significant (*P* < .05).

^d^ Asian: Asian American, Native Hawaiian, and Pacific Islander.

^e^ *P* value for the interaction between neighborhood disadvantage and race and ethnicity.

### Sensitivity Analysis

We conducted the following sensitivity analysis: using Fine and Gray models (eTables 6-7 in Supplement 1) and splitting the neighborhood disadvantage score into tertiles based on the national average (eTables 8-9 in Supplement 1). The findings were consistent with those from the main models.

## Discussion

In this national cohort study of 501 444 adults with ESKD and 95 068 KT candidates (2015-2021), residing in high-disadvantage neighborhoods was associated with a significantly lower likelihood of waitlisting and KT. The associations between neighborhood disadvantage and KT access were most pronounced among minoritized patients and those residing in the Western regions of the US. Residence in suburban or rural high-disadvantage neighborhoods was associated with especially low access to LDKT.

Our findings are consistent with previous literature,^6,33^ which found that racial and ethnic segregation, a form of neighborhood disadvantage, is associated with decreased access to waitlisting and KT. Moreover, other manifestations of neighborhood disadvantage, particularly socioeconomic and structural disadvantages, are associated with decreased access to LDKT and preemptive KT.^34,35^ We build upon these studies^6,33,34,35^ by examining various domains of neighborhood disadvantage, which allows us to capture the interconnected factors contributing to this inequality. We found that higher levels of disadvantages across the 9 neighborhood disadvantage score domains were associated with reduced access to waitlisting, LDKT, and preemptive KT. This may be because, within residential neighborhoods, disadvantage likely manifests through these domains and other systematic barriers that intersect to limit educational and employment opportunities,^8,34,35,36^ concentrate poverty,^8,19,34,35,37,38^ and restrict neighborhood resources,^8,19,34^ all of which can negatively influence health and reduce access to waitlisting and KT.

Additionally, we identified residence in a high-disadvantage neighborhood as a key driver of reduced access to waitlisting and KT. Residing in these neighborhoods also contributed to persistent racial and ethnic disparities in access to LDKT and preemptive KT. Specifically, Black and Hispanic individuals in high-disadvantage neighborhoods had a significantly lower likelihood of accessing KT compared with White individuals in low-disadvantage neighborhoods. It is likely that minoritized adults residing in these neighborhoods face multifactorial stressors, including unequal access to resources, substandard living conditions, high crime rates, and limited educational, employment, and health care access, that contribute to a higher prevalence of comorbidities.^6,16,17,34,39,40,41^ These interconnected conditions can also influence the development and progression of ESKD and ultimately reduce access to KT.^6^ For LDKT, in addition to the factors mentioned earlier, reduced social cohesion and weaker social networks may further limit access^6,8^ because living donors often come from the same communities as recipients and may be subject to similar conditions.^6,8^ In addition to material deprivation, neighborhoods affected by disadvantages may also foster mistrust of physicians and health care systems, reduce health-seeking behaviors, and ultimately make KT receipt more difficult.^6,17,18,33^ One study of predominantly Black, Hispanic, and Asian communities found that limited knowledge about the benefits of LDKT, concerns about donation risks, and mistrust of the health care system can reduce the availability of donors and pose challenges for both candidates and donors.^18^ For preemptive KT, in addition to the aforementioned factors, disparities may also be influenced by access to pre-ESKD nephrologic care, early transplant education, timely eligibility assessment, and referral, particularly among minoritized individuals with low income.^42^ Therefore, through material and social conditions, residence in high-disadvantage neighborhoods can exert a disproportionate impact on minoritized individuals, creating barriers in access to waitlisting and KT and exacerbating extant disparities.

Furthermore, we found regional- and urbanicity-related variations in the association of neighborhood disadvantage with access to waitlisting, LDKT, and preemptive KT. Residence in high-disadvantage neighborhoods was associated with reduced access, particularly in the Western regions, for all outcomes and in suburban or rural areas for LDKT. This finding may reflect regional differences in how neighborhood disadvantage manifests, contributing to health care inequities.^37,43,44,45^ States in the West face greater disadvantages in social and structural determinants of health (eg, limited access to care and healthy foods), which may in turn contribute to barriers to waitlisting and reduced access to KT.^17,37,46,47,48,49,50^ Additionally, suburban and rural residents have lower access to health care practitioners (primary care and specialists) and weaker referral networks,^19,22,51,52^ factors that may hinder timely access to waitlisting and KT, which requires coordinated and multidisciplinary care.^9,42,53^

Addressing the impact of neighborhood disadvantage on access to waitlisting and KT will require multifaceted interventions at both the local and national levels.^6,16,17,54^ This effort may involve multiple stakeholders,^6,16,17,54^ and interventions aimed at improving neighborhood investment (eg, built environment disadvantage, education disadvantage, employment, and housing instability, all of which are key components of neighborhood disadvantage) and promoting community development to counteract policies and practices that perpetuate neighborhood disadvantage.^6,16,17,54^ Community outreach programs that engage, educate, and support transplant candidates and potential donors in high-disadvantage neighborhoods are also critical.^6,16,17,54^

### Strengths and Limitations

Our study has several strengths, including the use of a large cohort and national registry data to examine the association between neighborhood disadvantage and access to waitlisting and KT. However, limitations exist. First, we used zip codes as a proxy for neighborhoods, which may have caused spatial misclassification and systemic bias.^55^ Nevertheless, zip codes are a commonly used proxy for defining neighborhoods.^56,57,58^ Second, it is unclear how long individuals resided in these neighborhoods, which may influence their cumulative exposure to stressors associated with high disadvantage. Third, due to data constraints, we were unable to assess when individuals were referred by nephrologists or other practitioners or when they were evaluated before being added to the waitlist. Fourth, we did not account for clustering or spatial dependency among ZCTAs and zip codes, which could be addressed with multilevel and spatial regression, respectively.

## Conclusions

In this cohort study of adults with ESKD and KT candidates, residence in a disadvantaged neighborhood was associated with reduced access to waitlisting and KT. These findings further suggest that neighborhood disadvantage may contribute to persistent racial and ethnic disparities in access to LDKT and preemptive KT. Urgent, multifaceted interventions (eg, community outreach programs and patient navigators)^54,59^ are needed to dismantle the structural barriers that contribute to high levels of neighborhood disadvantage. Future research and collaborative efforts should develop and evaluate strategies targeting upstream drivers of neighborhood disadvantage that contribute to inequities in access to waitlisting and KT.

## Notes

Supplement 1. **eTable 1.** The domains/components of the neighborhood disadvantage score **eTable 2.** Residential neighborhood disadvantage and time to waitlisting among adults (aged ≥18) with end- stage kidney disease initiating dialysis, and time to live-donor kidney transplantation (LDKT) and preemptive kidney transplantation (KT) among KT candidates (aged ≥18) stratified by US region, and urbanicity (2015-2021; N=501,444) **eTable 3.** Characteristics of the listing population (KT candidates; aged ≥18) from 2015-2021, stratified by residential neighborhood disadvantage score (N=95,068) **eTable 4.** Residential neighborhood disadvantage and time to kidney transplantation (KT) among KT candidates (aged ≥18), stratified by race and ethnicity, US region, and urbanicity (2015-2021; N=95,068) **eTable 5.** Components of residential neighborhood disadvantage and time to kidney transplantation (KT) among KT candidates (aged ≥18) (2015-2021) **eTable 6.** [Sensitivity Analysis – Competing Risk Model] Residential neighborhood disadvantage and time to listing among adults (aged ≥18) with end-stage kidney disease initiating dialysis, stratified by race and ethnicity, US region, and urbanicity (2015-2021; N=501,444) **eTable 7.** [Sensitivity Analysis – Competing Risk Model] Residential neighborhood disadvantage and time to live-donor kidney transplantation (LDKT) and preemptive kidney transplantation (KT) among KT candidates (aged ≥18), stratified by race and ethnicity, US region, and urbanicity (2015-2021; N=95,068) **eTable 8.** [Sensitivity Analysis – National Tertiles] Residential neighborhood disadvantage and time to listing among adults (aged ≥18) with end-stage kidney disease initiating dialysis, stratified by race and ethnicity, US region, and urbanicity **eTable 9.** [Sensitivity Analysis – National Tertiles] Residential neighborhood disadvantage and time to live- donor kidney transplantation (LDKT) and preemptive kidney transplantation (KT) among KT candidates (aged ≥18), stratified by race and ethnicity, US region, and urbanicity (2015-2021; N=95,068) **eFigure 1.** Residential neighborhood disadvantage and time to A) live-donor kidney transplantation (LDKT), and B) preemptive kidney transplantation (2015-2021)

Supplement 2.
Data Sharing Statement
