# Supplementary material for: Residential Neighborhood Disadvantage and Access to Kidney Transplantation
Source: JAMA Netw Open. 2025 Dec 30;8(12):e2549679. doi: 10.1001/jamanetworkopen.2025.49679 (PMC12754678; doi:10.1001/jamanetworkopen.2025.49679)
Supplement: Supplement 1. — eTable 1. The domains/components of the neighborhood disadvantage score eTable 2. Residential neighborhood disadvantage and time to waitlisting among adults (aged ≥18) with end- stage kidney disease initiating dialysis, and time to live-donor kidney transplantation (LDKT) and preemptive kidney transplantation (KT) among KT candidates (aged ≥18) stratified by US region, and urbanicity (2015-2021; N=501,444) eTable 3. Characteristics of the listing population (KT candidates; aged ≥18) from 2015-2021, stratified by residential neighborhood disadvantage score (N=95,068) eTable 4. Residential neighborhood disadvantage and time to kidney transplantation (KT) among KT candidates (aged ≥18), stratified by race and ethnicity, US region, and urbanicity (2015-2021; N=95,068) eTable 5. Components of residential neighborhood disadvantage and time to kidney transplantation (KT) among KT candidates (aged ≥18) (2015-2021) eTable 6. [Sensitivity Analysis – Competing Risk Model] Residential neighborhood disadvantage and time to listing among adults (aged ≥18) with end-stage kidney disease initiating dialysis, stratified by race and ethnicity, US region, and urbanicity (2015-2021; N=501,444) eTable 7. [Sensitivity Analysis – Competing Risk Model] Residential neighborhood disadvantage and time to live-donor kidney transplantation (LDKT) and preemptive kidney transplantation (KT) among KT candidates (aged ≥18), stratified by race and ethnicity, US region, and urbanicity (2015-2021; N=95,068) eTable 8. [Sensitivity Analysis – National Tertiles] Residential neighborhood disadvantage and time to listing among adults (aged ≥18) with end-stage kidney disease initiating dialysis, stratified by race and ethnicity, US region, and urbanicity eTable 9. [Sensitivity Analysis – National Tertiles] Residential neighborhood disadvantage and time to live- donor kidney transplantation (LDKT) and preemptive kidney transplantation (KT) among KT candidates (aged ≥18), stratified by race and ethnicity, US r [file jamanetwopen-e2549679-s001.pdf]

Supplemental Online Content

Li Y, Menon G, Kim B, et al. Residential neighborhood disadvantage and access to kidney transplantation. *JAMA Netw Open*. 2025;8(12):e2549679.
doi:10.1001/jamanetworkopen.2025.49679

eTable 1. The domains/components of the neighborhood disadvantage score

eTable 2. Residential neighborhood disadvantage and time to waitlisting among adults (aged ≥18) with end-stage kidney disease initiating dialysis, and time to live-donor kidney transplantation (LDKT) and preemptive kidney transplantation (KT) among KT candidates (aged ≥18) stratified by US region, and urbanicity (2015-2021; N=501,444)

eTable 3. Characteristics of the listing population (KT candidates; aged ≥18) from 2015-2021, stratified by residential neighborhood disadvantage score (N=95,068)

eTable 4. Residential neighborhood disadvantage and time to kidney transplantation (KT) among KT candidates (aged ≥18), stratified by race and ethnicity, US region, and urbanicity (2015-2021; N=95,068)

eTable 5. Components of residential neighborhood disadvantage and time to kidney transplantation (KT) among KT candidates (aged ≥18) (2015-2021)

eTable 6. [Sensitivity Analysis – Competing Risk Model] Residential neighborhood disadvantage and time to listing among adults (aged ≥18) with end-stage kidney disease initiating dialysis, stratified by race and ethnicity, US region, and urbanicity (2015-2021; N=501,444)

eTable 7. [Sensitivity Analysis – Competing Risk Model] Residential neighborhood disadvantage and time to live-donor kidney transplantation (LDKT) and preemptive kidney transplantation (KT) among KT candidates (aged ≥18), stratified by race and ethnicity, US region, and urbanicity (2015-2021; N=95,068)

eTable 8. [Sensitivity Analysis – National Tertiles] Residential neighborhood disadvantage and time to listing among adults (aged ≥18) with end-stage kidney disease initiating dialysis, stratified by race and ethnicity, US region, and urbanicity

eTable 9. [Sensitivity Analysis – National Tertiles] Residential neighborhood disadvantage and time to live-donor kidney transplantation (LDKT) and preemptive kidney transplantation (KT) among KT candidates (aged ≥18), stratified by race and ethnicity, US region, and urbanicity (2015-2021; N=95,068)

eFigure 1. Residential neighborhood disadvantage and time to A) live-donor kidney transplantation (LDKT), and B) preemptive kidney transplantation (2015-2021)

This supplemental material has been provided by the authors to give readers additional information about their work.

© 2025 Li Y et al. *JAMA Network Open.*

eTable 1. The domains/components of the neighborhood disadvantage score

Domains	Variables
Built environment disadvantage	Building vacancy rate, mobile homes, no internet access, cancer risk, low food access for SNAP recipients
Criminal injustice	Pretrail jail rate, total jail rate, law enforcement personnel per capita
Education disadvantage	Bachelor's degree or higher, high school diploma, per pupil spending (school district)
Unemployment	Unemployed, white-collar occupation, retail job availability
Housing instability	Housing units without telephone, housing units without plumbing, crowding, group quarters, foreclosure risk, eviction rate
Poverty	Below 100% FPL, below 200% FPL, public assistance, family income, per capita income
Social fragmentation	Changed address in last year, single-parent households, income gap, residential segregation
Transportation barrier	Carpooled to work, no access to motor vehicle, took public transit to work, biked to work, walked to work, transportation cost burden, median-income family
Wealth inequality	Aggregate home value, median real estate taxes paid, median home value, median gross rent, median monthly mortgage, owner-occupied homes

The neighborhood disadvantage score was created by Dr. Dyer, and the data were obtained from https://www.sreindex.com/. As increasing values of the nine domains indicated more disadvantages, the naming convention was revised to reflect the appropriate interpretation of the domains and to improve clarity.

© 2025 Li Y et al. *JAMA Network Open.*

eTable 2. Residential neighborhood disadvantage and time to waitlisting among adults (aged≥18) with end-stage kidney disease initiating dialysis, and time to live-donor kidney transplantation (LDKT) and preemptive kidney transplantation (KT) among KT candidates (aged≥18) stratified by US region, and urbanicity (2015-2021; N=501,444)

	Adjusted Hazard Ratio (aHR) (95% Confidence Interval) Residential Neighborhood Disadvantage Score[a]		
	**Low**	**Medium**	**High**
**Listed (N=501,444)[b]**			
**Overall**	Reference	0.83 (0.81-0.84)[f]	0.71 (0.69-0.72)[f]
**US Region**			
Northeast	Reference	0.88 (0.84-0.92)[f]	0.86 (0.81-0.91)[f]
Midwest	0.85 (0.82-0.89)[f]	0.70 (0.68-0.73)[f]	0.57 (0.55-0.60)[f]
South	0.85 (0.82-0.88)[f]	0.72 (0.70-0.74)[f]	0.60 (0.58-0.62)[f]
West	0.74 (0.71-0.76)[f]	0.62 (0.60-0.65)[f]	0.57 (0.55-0.60)[f]
p-value for interaction[c]	.001[f]	<.001[f]	0.01[f]
**Urbanicity[d]**			
HDU	Reference	0.84 (0.82-0.87)[f]	0.73 (0.71-0.75)[f]
Suburban	1.08 (1.05-1.11)[f]	0.88 (0.86-0.91)[f]	0.73 (0.70-0.75)[f]
Rural	1.01 (0.96-1.06)	0.85 (0.81-0.88)[f]	0.73 (0.70-0.76)[f]
Small town	0.90 (0.84-0.97)[f]	0.80 (0.76-0.83)[f]	0.71 (0.68-0.74)[f]
p-value for interaction[c]	.005[f]	<.001[f]	0.59
**LDKT (N=95,068)[e]**			
Overall	Reference	0.82 (0.78-0.85)[f]	0.65 (0.62-0.69)[f]
**US Region**			
Northeast	Reference	0.78 (0.71-0.86)[f]	0.59 (0.50-0.69)[f]
Midwest	1.05 (0.98-1.13)	0.95 (0.88-1.03)	0.79 (0.70-0.88)[f]
South	1.15 (1.07-1.23)[f]	0.85 (0.80-0.92)[f]	0.67 (0.63-0.73)[f]
West	0.77 (0.71-0.83)[f]	0.58 (0.53-0.64)[f]	0.43 (0.38-0.49)[f]
p-value for interaction[c]	<.001[f]	<.001[f]	.001[f]
**Urbanicity[d]**			
HDU	Reference	0.76 (0.71-0.81)[f]	0.60 (0.55-0.66)[f]
Suburban	1.04 (0.99-1.10)	0.81 (0.76-0.87)[f]	0.72 (0.66-0.78)[f]
Rural	1.18 (1.08-1.28)[f]	1.00 (0.92-1.09)	0.69 (0.62-0.77)[f]
Small town	0.93 (0.81-1.08)	0.96 (0.87-1.06)	0.71 (0.64-0.79)[f]
p-value for interaction[c]	<.001[f]	<.001[f]	<.001[f]
**Preemptive KT (N=95,068)[e]**			
Overall	Reference	0.75 (0.72-0.79)[f]	0.62 (0.58-0.67)[f]
**US Region**			
Northeast	Reference	0.67 (0.60-0.76)[f]	0.58 (0.47-0.72)[f]
Midwest	1.12 (1.04-1.21)[f]	0.87 (0.80-0.95)[f]	0.69 (0.59-0.79)[f]
South	0.98 (0.90-1.06)	0.74 (0.68-0.81)[f]	0.62 (0.56-0.67)[f]
West	0.75 (0.69-0.82)[f]	0.57 (0.50-0.64)[f]	0.45 (0.38-0.54)[f]

© 2025 Li Y et al. *JAMA Network Open.*

p-value for interaction[c]	<.001	<.001[f]	<.001[f]
**Urbanicity**[d]			
HDU	Reference	0.70 (0.64-0.77)[f]	0.59 (0.52-0.66)[f]
Suburban	1.10 (1.03-1.17)[f]	0.79 (0.72-0.85)[f]	0.66 (0.59-0.75)[f]
Rural	1.12 (1.02-1.24)[f]	0.80 (0.72-0.89)[f]	0.70 (0.61-0.79)[f]
Small town	1.03 (0.88-1.20)	1.03 (0.93-1.15)	0.71 (0.62-0.81)[f]
p-value for interaction[c]	<.001[f]	<.001[f]	0.59

Abbreviation: HDU=high density urban

[a]Neighborhood disadvantage score: nine domains (built environment disadvantage, criminal injustice, education disadvantage, unemployment, housing instability, poverty, social fragmentation, transportation barrier, and wealth inequality). As increasing values of the nine domains indicated more disadvantages, the naming convention was revised to ref lect the appropriate interpretation of the domains and to improve clarity. https://www.sreindex.com/

[b]Cause-specific hazards models adjusted for year of dialysis initiation, age, sex, cause of ESKD, employment status, body mass index (BMI), nephrology care, comorbidities (cancer, hypertension, diabetes, peripheral vascular disease, atherosclerotic heart disease, congestive heart failure, chronic obstructive pulmonary disease, drug use, alcohol use, tobacco use), and f unctional status.

[c]P-value for the interaction between neighborhood disadvantage and US region; neighborhood disadvantage and urbanicity

[d]Modified Rural-Urban Commuting Area [RUCA] Codes defined by US Department of Agriculture; high-density urban, suburban, rural, and small town. https://www.sciencedirect.com/science/article/pii/S2590291122000043

[e]Cause-specific hazards models adjusted for year of listing, age at listing, sex, cause of ESKD, body mass index (BMI), blood type, peak reactivity antibody, comorbidities (cancer, hypertension, diabetes, peripheral vascular disease, atherosclerotic heart disease, congestive heart failure, chronic obstructive pulmonary disease, drug use, alcohol use, tobacco use), and functional status.

[f]Associations that are statistically significant ($p<0.05$)

© 2025 Li Y et al. *JAMA Network Open.*

eTable 3. Characteristics of the listing population (KT candidates; aged ≥18) from 2015-2021, stratified by residential neighborhood disadvantage score (N=95,068)

Patient Characteristics, N (%)	Total N=95,068	Neighborhood Disadvantage Score[a] Low N=37,982	Medium N=30,368	High N=26,718
**Age in years, mean (SD)**	53.7 (13.0)	52.1 (12.6)	55.2 (13.0)	53.3 (13.0)
**Age group**				
18-34	9407 (9.9)	3261 (8.6)	3243 (10.7)	2903 (10.9)
35-49	22845 (24.0)	7985 (21.0)	7433 (24.5)	7427 (27.8)
50-64	41298 (43.4)	16405 (43.2)	13133 (43.2)	11760 (44.0)
≥ 65	21518 (22.6)	10331 (27.2)	6559 (21.6)	4628 (17.3)
**Sex**				
Male	60328 (63.5)	24865 (65.5)	19207 (63.2)	16256 (60.8)
Female	34740 (36.5)	13117 (34.5)	11161 (36.8)	10462 (39.2)
**Race/Ethnicity**				
Asian	6956 (7.3)	4476 (11.8)	1774 (5.8)	706 (2.6)
Black	25215 (26.5)	6291 (16.6)	7626 (25.1)	11298 (42.3)
Hispanic	15685 (16.5)	4123 (10.9)	5496 (18.1)	6066 (22.7)
White	47212 (49.7)	23092 (60.8)	15472 (50.9)	8648 (32.4)
**BMI in kg/m^2, mean (SD)**	29.5 (6.6)	30.1 (6.6)	29.0 (6.5)	29.7 (6.6)
**BMI group**				
≤25	24722 (26.0)	11011 (29.0)	7586 (25.0)	6125 (22.9)
26-30	29667 (31.2)	12076 (31.8)	9380 (30.9)	8211 (30.7)
>30	40679 (42.8)	14895 (39.2)	13402 (44.1)	12382 (46.3)
**Blood Type**				
A	31590 (33.2)	13223 (34.8)	10282 (33.9)	8085 (30.3)
B	14173 (14.9)	5736 (15.1)	4305 (14.2)	4132 (15.5)
AB	3777 (4.0)	1680 (4.4)	1177 (3.9)	920 (3.4)
O	45528 (47.9)	17343 (45.7)	14604 (48.1)	13581 (50.8)
**Cause of ESKD**				
Diabetes mellitus	35137 (37.0)	13069 (34.4)	11334 (37.3)	10734 (40.2)
Hypertension	25701 (27.0)	8991 (23.7)	7973 (26.3)	8737 (32.7)
Glomerulonephritis	15540 (16.3)	6978 (18.4)	5019 (16.5)	3543 (13.3)
Other	18690 (19.7)	8944 (23.5)	6042 (19.9)	3704 (13.9)
**Comorbidities**				

© 2025 Li Y et al. *JAMA Network Open.*

Cancer	3097 (3.3)	1507 (4.0)	955 (3.1)	635 (2.4)
Peripheral vascular disease	3262 (3.4)	1319 (3.5)	1038 (3.4)	905 (3.4)
Cerebrovascular disease	3372 (3.5)	1266 (3.3)	1098 (3.6)	1008 (3.8)
Atherosclerotic heart disease	5533 (5.8)	2468 (6.5)	1736 (5.7)	1329 (5.0)
CHF	10330 (10.9)	3826 (10.1)	3308 (10.9)	3196 (12.0)
COPD	1892 (2.0)	702 (1.8)	646 (2.1)	544 (2.0)
Drug use	450 (0.5)	147 (0.4)	147 (0.5)	156 (0.6)
Alcohol use	1173 (1.2)	497 (1.3)	384 (1.3)	292 (1.1)
Tobacco use	3226 (3.4)	1027 (2.7)	1097 (3.6)	1102 (4.1)
Functional Impairment	2987 (3.1)	1060 (2.8)	966 (3.2)	961 (3.6)
Institutionalized	520 (0.5)	221 (0.6)	168 (0.6)	131 (0.5)
**Calculated PRA>80%**	6619 (7.0)	2101 (5.5)	2111 (7.0)	2407 (9.0)
**Urbanicity[b]**				
High-density urban	38746 (40.8)	16972 (44.7)	11484 (37.8)	10290 (38.5)
Suburban	34686 (36.5)	15836 (41.7)	11075 (36.5)	7775 (29.1)
Rural	12533 (13.2)	3831 (10.1)	4320 (14.2)	4382 (16.4)
Small town	9103 (9.6)	1343 (3.5)	3489 (11.5)	4271 (16.0)

Abbrevations: ESKD=End-Stage Kidney Disease; SD = standard deviation; COPD = Chronic Obstructive Pulmonary Disease; CHF = Congestive Heart Failure; BMI=Body Mass Index;
PRA= panel reactive antibody

[a]Neighborhood disadvantage score: nine domains (built environment disadvantage, criminal injustice, education disadvantage, unemployment, housing instability, poverty, social fragmentation, transportation barrier, and wealth inequality). As increasing values of the nine domains indicated more disadvantages, the naming convention was revised to ref lect the appropriate interpretation of the domains and to improve clarity. https://www.sreindex.com/

[b]This classification modified the original 2010 Rural-Urban Commuting Area Codes (RUCA) Codes defined by United States Department of Agriculture (USDA). https://www.sciencedirect.com/science/article/pii/S2590291122000043

© 2025 Li Y et al. *JAMA Network Open.*

eTable 4. Residential neighborhood disadvantage and time to kidney transplantation (KT) among KT candidates (aged≥18), stratified by race and ethnicity, US region, and urbanicity (2015-2021; N=95,068)

	Adjusted Hazard Ratio (aHR) (95% Confidence Interval) Residential Neighborhood Disadvantage Score[a]		
	**Low**	**Medium**	**High**
**Overall**	Reference	0.96 (0.94-0.99)[e]	0.89 (0.87-0.92)[e]
**Race/Ethnicity**[b]			
Asian	0.54 (0.52-0.57)[e]	0.60 (0.56-0.65)[e]	0.57 (0.51-0.64)[e]
Black	0.65 (0.62-0.67)[e]	0.63 (0.61-0.66)[e]	0.60 (0.58-0.62)[e]
Hispanic	0.71 (0.67-0.74)[e]	0.67 (0.64-0.70)[e]	0.62 (0.59-0.65)[e]
White	Reference	0.95 (0.93-0.98)[e]	0.87 (0.84-0.90)[e]
p-value for interaction[c]	<.001[e]	.007[e]	.04[e]
Asian	Reference	1.10 (1.07-1.14)[e]	1.02 (0.98-1.07)
Black	Reference	0.87 (0.84-0.90)[e]	0.86 (0.83-0.89)[e]
Hispanic	Reference	0.83 (0.79-0.87)[e]	0.82 (0.79-0.86)[e]
White	Reference	0.95 (0.93-0.98)[e]	0.87 (0.84-0.90)[e]
p-value for interaction[c]		<.001[e]	<.001[e]
**US Region**			
Northeast	Reference	0.87 (0.82-0.91)[e]	0.80 (0.75-0.87)[e]
Midwest	1.36 (1.31-1.41)[e]	1.36 (1.31-1.42)[e]	1.31 (1.24-1.38)[e]
South	1.20 (1.16-1.25)[e]	1.11 (1.07-1.15)[e]	1.00 (0.96-1.03)
West	0.88 (0.85-0.92)[e]	0.79 (0.75-0.83)[e]	0.67 (0.63-0.72)[e]
p-value for interaction[c]	<.001[e]	<.001[e]	<.001[e]
**Urbanicity**[d]			
HDU	Reference	0.94 (0.91-0.98)[e]	0.92 (0.88-0.95)[e]
Suburban	1.12 (1.08-1.15)[e]	1.02 (0.98-1.05)	0.95 (0.91-1.00)[e]
Rural	1.15 (1.10-1.21)[e]	1.08 (1.03-1.13)[e]	0.97 (0.92-1.02)
Small town	1.06 (0.98-1.15)	1.26 (1.19-1.32)[e]	0.96 (0.92-1.02)
p-value for interaction[c]	.71	<.001[e]	.69

Abbreviation: HDU=high density urban

Cause-specific hazards models adjusted for year of listing, age at listing, race and ethnicity, sex, cause of ESKD, body mass index (BMI), blood type, peak reactivity antibody, comorbidities (cancer, hypertension, diabetes, peripheral vascular disease, atherosclerotic heart disease, congestive heart failure, chronic obstructive pulmonary disease, drug use, alcohol use, tobacco use), and functional status.

[a]Neighborhood disadvantage score: nine domains (built environment disadvantage, criminal injustice, education disadvantage, unemployment, housing instability, poverty, social fragmentation, transportation barrier, and wealth inequality). As increasing values of the nine domains indicated more disadvantages, the naming convention was revised to reflect the appropriate interpretation of the domains and to improve clarity. https://www.sreindex.com/

[b]Race and ethnicity: non-Hispanic White, non-Hispanic Black, Hispanic and non-Hispanic Asian (Asian American, Native Hawaiian, and Pacific Islander).

[c]P-value for the interaction between neighborhood disadvantage score and race and ethnicity; neighborhood disadvantage score and US region; neighborhood disadvantage score and urbanicity

[d]Modified Rural-Urban Commuting Area [RUCA] Codes defined by US Department of Agriculture; high-density urban, suburban, rural, and small town.

[e]Associations that are statistically significant (p<0.05)

© 2025 Li Y et al. *JAMA Network Open.*

eTable 5. Components of residential neighborhood disadvantage and time to kidney transplantation (KT) among KT candidates (aged≥18) (2015-2021)

	Adjusted Hazard Ratio (aHR) (95% Confidence Interval) N=95,068
**Neighborhood Disadvantage Domain**	
Built Environment Disadvantage[a]	**0.96 (0.95-0.97)**
Criminal Injustice[b]	**0.97 (0.96-0.98)**
Education Disadvantage[c]	**0.94 (0.93-0.95)**
Unemployment[d]	**0.94 (0.93-0.95)**
Housing Instability[e]	**0.91 (0.90-0.93)**
Poverty[f]	**0.92 (0.91-0.93)**
Social Fragmentation[g]	**0.94 (0.92-0.95)**
Transportation Barrier[h]	**1.04 (1.03-1.05)**
Wealth Inequality[i]	**1.04 (1.02-1.05)**

[*]Neighborhood disadvantage score: nine domains (built environment disadvantage, criminal injustice, education disadvantage, unemployment, housing instability, poverty, social fragmentation, transportation barrier, and wealth inequality). As increasing values of the nine domains indicated more disadvantages, the naming convention was revised to ref lect the appropriate interpretation of the domains and to improve clarity. https://www.sreindex.com/. Each domain's measures were standardized.

[a]Built environment disadvantage: building vacancy rate, mobile home, no internet access (American Community Survey 5-year estimates 2015-2019); Cancer risk (Environmental Protection Agency, Air Toxics Screening Assessment), low food access for SNAP recipients (Department of Agriculture, Food Access Research Atlas 2021)

[b]Criminal injustice: Pretrial jail rate, total jail rate, law enf orcement personnel per capita (municipality)

[c]Education disadvantage: Bachelor's degree or higher, high school diploma (American Community Survey 5-year estimates 2015-2019); per pupil spending (school district)

[d]Unemployment: Unemployed, white-collar occupation; Retail job availability (stratify by age)

[e]Housing instability: Housing units without telephone, housing units without plumbing, crowding, group quarters; foreclosure risk; eviction rate

[f]Poverty: Below 100% FPL, below 200% FPL, public assistance, f amily income, per capita income; supplemental poverty measure

[g]Social f ragmentation: Changed address in last year, single-parent households, income gap; residential segregation

[h]Transportation barrier: Carpooled to work, no access to a motor vehicle, took public transit to work, biked to work, walked to work; Transportation cost burden, median income f amily

[i]Wealth inequality: Aggregate home value, median real estate taxes paid, median home value, median gross rent, median monthly mortgage, owner-occupied homes

Cause-specific hazards models adjusted for year of listing, age at listing, race and ethnicity, sex, cause of ESKD, body mass index (BMI), blood type, peak reactivity antibody, comorbidities (cancer, hypertension, diabetes, peripheral vascular disease, atherosclerotic heart disease, congestive heart failure, chronic obstructive pulmonary disease, drug use, alcohol use, tobacco use), and f unctional status.

© 2025 Li Y et al. *JAMA Network Open.*

eTable 6. [Sensitivity Analysis – Competing Risk Model] Residential neighborhood disadvantage and time to listing among adults (aged≥18) with end-stage kidney disease initiating dialysis, stratified by race and ethnicity, US region, and urbanicity (2015-2021; N=501,444)

Listed (N=501,444)	Adjusted Hazard Ratio (aHR) (95% Confidence Interval) Residential Neighborhood Disadvantage Score[a]		
	Low	Medium	High
**Overall**	Reference	0.82 (0.81-0.84)[e]	0.70 (0.68-0.71)[e]
**Race/Ethnicity**[b]			
Asian	1.21 (1.16-1.26)[e]	1.05 (0.99-1.11)	0.91 (0.83-0.99)[e]
Black	1.03 (0.99-1.06)	0.88 (0.85-0.91)[e]	0.71 (0.69-0.72)[e]
Hispanic	1.18 (1.13-1.22)[e]	1.00 (0.97-1.04)	0.93 (0.90-0.96)[e]
White	Reference	0.80 (0.78-0.82)[e]	0.67 (0.65-0.69)[e]
p-value for interaction[c]	<.001[e]	<.001[e]	<.001[e]
**US Region**			
Northeast	Reference	0.88 (0.84-0.92)[e]	0.86 (0.81-0.91)[e]
Midwest	0.85 (0.82-0.89)[e]	0.70 (0.67-0.73)[e]	0.57 (0.55-0.60)[e]
South	0.84 (0.81-0.88)[e]	0.71 (0.69-0.73)[e]	0.59 (0.57-0.61)[e]
West	0.74 (0.71-0.77)[e]	0.62 (0.60-0.65)[e]	0.57 (0.54-0.60)[e]
p-value for interaction[c]	.002[e]	<.001[e]	.014[e]
**Urbanicity**[d]			
HDU	Reference	0.84 (0.82-0.86)[e]	0.73 (0.71-0.75)[e]
Suburban	1.08 (1.05-1.11)[e]	0.88 (0.85-0.90)[e]	0.72 (0.69-0.74)[e]
Rural	1.00 (0.96-1.05)	0.83 (0.80-0.87)[e]	0.72 (0.69-0.75)[e]
Small town	0.89 (0.82-0.96)[e]	0.79 (0.76-0.83)[e]	0.70 (0.67-0.72)[e]
p-value for interaction[c]	<.001[e]	<.001[e]	.105

Abbreviation: HDU=high density urban

Fine and Gray Subdistribution hazard model (death as competing risk) adjusted for year of dialysis initiation, age, race and ethnicity, sex, cause of ESKD, employment status, body mass index (BMI), nephrology care, comorbidities (cancer, hypertension, diabetes, peripheral vascular disease, atherosclerotic heart disease, congestive heart failure, chronic obstructive pulmonary disease, drug use, alcohol use, tobacco use), and functional status.

[a]Neighborhood disadvantage score: nine domains (built environment disadvantage, criminal injustice, education disadvantage, unemployment, housing instability, poverty, social fragmentation, transportation barrier, and wealth inequality). As increasing values of the nine domains indicated more disadvantages, the naming convention was revised to reflect the appropriate interpretation of the domains and to improve clarity. https://www.sreindex.com/

[b]Race and ethnicity: non-Hispanic White, non-Hispanic Black, Hispanic and non-Hispanic Asian (Asian American, Native Hawaiian, and Pacific Islander).

[c]P-value for the interaction between neighborhood disadvantage score and race and ethnicity; neighborhood disadvantage score and US region; neighborhood disadvantage score and urbanicity

[d]Modified Rural-Urban Commuting Area [RUCA] Codes defined by US Department of Agriculture; high-density urban, suburban, rural, and small town. https://www.sciencedirect.com/science/article/pii/S2590291122000043

[e]Associations that are statistically significant (p<0.05)

© 2025 Li Y et al. *JAMA Network Open.*

eTable 7. [Sensitivity Analysis – Competing Risk Model] Residential neighborhood disadvantage and time to live-donor kidney transplantation (LDKT) and preemptive kidney transplantation (KT) among KT candidates (aged≥18), stratified by race and ethnicity, US region, and urbanicity (2015-2021; N=95,068)

	Adjusted Hazard Ratio (aHR) (95% Confidence Interval) Residential Neighborhood Disadvantage Score[a]					
	LDKT			Preemptive KT		
	**Low**	**Medium**	**High**	**Low**	**Medium**	**High**
**Overall**	Reference	0.81 (0.78-0.85)[e]	0.65 (0.62-0.68)[e]	Reference	0.75 (0.71-0.79)[e]	0.62 (0.58-0.66)[e]
**Race/Ethnicity**[b]						
Asian	0.44 (0.40-0.49)[e]	0.37 (0.31-0.44)[e]	0.25 (0.18-0.35)[e]	0.39 (0.34-0.44)[e]	0.31 (0.25-0.39)[e]	0.20 (0.13-0.32)[e]
Black	0.49 (0.45-0.53)[e]	0.32 (0.29-0.36)[e]	0.23 (0.21-0.25)[e]	0.29 (0.26-0.34)[e]	0.25 (0.22-0.29)[e]	0.22 (0.20-0.25)[e]
Hispanic	0.69 (0.63-0.76)[e]	0.55 (0.51-0.60)[e]	0.51 (0.47-0.56)[e]	0.51 (0.45-0.57)[e]	0.35 (0.31-0.40)[e]	0.25 (0.21-0.29)[e]
White	Reference	0.84 (0.80-0.88)[e]	0.68 (0.64-0.73)[e]	Reference	0.74 (0.70-0.79)[e]	0.63 (0.58-0.68)[e]
p-value for interaction[c]	<.001[e]	<.001[e]	<.001[e]	<.001[e]	.001[e]	<.001[e]
**US Region**						
Northeast	Reference	0.78 (0.71-0.86)[e]	0.59 (0.50-0.69)[e]	Reference	0.67 (0.60-0.76)[e]	0.58 (0.47-0.72)[e]
Midwest	1.05 (0.98-1.13)	0.95 (0.88-1.03)	0.78 (0.70-0.88)[e]	1.12 (1.04-1.21)[e]	0.87 (0.79-0.95)[e]	0.69 (0.59-0.79)[e]
South	1.14 (1.07-1.22)[e]	0.85 (0.79-0.91)[e]	0.67 (0.62-0.72)[e]	0.98 (0.90-1.06)	0.74 (0.68-0.80)[e]	0.61 (0.56-0.67)[e]
West	0.77 (0.71-0.83)[e]	0.58 (0.52-0.63)[e]	0.43 (0.37-0.49)[e]	0.76 (0.69-0.82)[e]	0.56 (0.50-0.63)[e]	0.45 (0.38-0.54)[e]
p-value for interaction[c]	<.001[e]	<.001[e]	<.001[e]	<.001[e]	<.001[e]	<.001[e]
**Urbanicity**[d]						
HDU	Reference	0.75 (0.70-0.81)[e]	0.60 (0.55-0.65)[e]	Reference	0.70 (0.64-0.77)[e]	0.58 (0.52-0.66)[e]
Suburban	1.04 (0.98-1.10)	0.81 (0.76-0.87)[e]	0.71 (0.65-0.78)[e]	1.10 (1.03-1.17)[e]	0.78 (0.72-0.85)[e]	0.66 (0.59-0.75)[e]
Rural	1.17 (1.08-1.28)[e]	0.99 (0.90-1.08)	0.68 (0.62-0.76)[e]	1.12 (1.02-1.23)[e]	0.79 (0.71-0.88)[e]	0.69 (0.61-0.79)[e]
Small town	0.93 (0.80-1.07)	0.95 (0.87-1.05)	0.71 (0.63-0.79)[e]	1.03 (0.88-1.20)	1.03 (0.92-1.14)	0.71 (0.62-0.81)[e]
p-value for interaction[c]	<.001[e]	0.11	<.001[e]	0.65	.001[e]	0.86

Abbreviation: HDU=high density urban

Fine and Gray Subdistribution hazard model (death as competing risk) adjusted for year of listing, age at listing, race and ethnicity, sex, cause of ESKD, body mass index (BMI), blood type, peak reactivity antibody, comorbidities (cancer, hypertension, diabetes, peripheral vascular disease, atherosclerotic heart disease, congestive heart failure, chronic obstructive pulmonary disease, drug use, alcohol use, tobacco use), and functional status.

[a]Neighborhood disadvantage score: nine domains (built environment disadvantage, criminal injustice, education disadvantage, unemployment, housing instability, poverty, social fragmentation, transportation barrier, and wealth inequality). As increasing values of the nine domains indicated more disadvantages, the naming convention was revised to reflect the appropriate interpretation of the domains and to improve clarity. https://www.sreindex.com/

[b]Race and ethnicity: non-Hispanic White, non-Hispanic Black, Hispanic and non-Hispanic Asian (Asian American, Native Hawaiian, and Pacific Islander).

[c]P-value for the interaction between neighborhood disadvantage score and race and ethnicity; neighborhood disadvantage score and US region; neighborhood disadvantage score and urbanicity

[d]Modified Rural-Urban Commuting Area [RUCA] Codes defined by US Department of Agriculture; high-density urban, suburban, rural, and small town. https://www.sciencedirect.com/science/article/pii/S2590291122000043

[e]Associations that are statistically significant (p<0.05)

© 2025 Li Y et al. *JAMA Network Open.*

eTable 8. [Sensitivity Analysis – National Tertiles] Residential neighborhood disadvantage and time to listing among adults (aged≥18) with end-stage kidney disease initiating dialysis, stratified by race and ethnicity, US region, and urbanicity (2015-2021; N=501,444)

Listed (N=501,444)	Adjusted Hazard Ratio (aHR) (95% Confidence Interval) Residential Neighborhood Disadvantage Score[a]		
	**Low**	**Medium**	**High**
Overall	Reference	0.84 (0.82-0.85)[e]	0.70 (0.69-0.72)[e]
Race/Ethnicity[b]			
Asian	1.12 (1.07-1.17)[e]	1.01 (0.95-1.06)	0.87 (0.81-0.94)[e]
Black	0.97 (0.94-1.01)	0.85 (0.82-0.87)[e]	0.68 (0.66-0.70)[e]
Hispanic	1.11 (1.07-1.16)[e]	0.96 (0.92-0.99)[e]	0.88 (0.85-0.90)[e]
White	Reference	0.81 (0.79-0.83)[e]	0.67 (0.65-0.69)[e]
p-value for interaction[c]	<.001[e]	<.001[e]	<.001[e]
US Region			
Northeast	Reference	0.89 (0.85-0.93)[e]	0.86 (0.82-0.91)[e]
Midwest	0.87 (0.84-0.91)[e]	0.72 (0.69-0.74)[e]	0.59 (0.56-0.61)[e]
South	0.87 (0.84-0.90)[e]	0.75 (0.72-0.77)[e]	0.61 (0.59-0.62)[e]
West	0.74 (0.72-0.77)[e]	0.63 (0.60-0.65)[e]	0.59 (0.56-0.61)[e]
p-value for interaction[c]	<.001[e]	<.001[e]	.002[e]
Urbanicity[d]			
HDU	Reference	0.84 (0.82-0.87)[e]	0.73 (0.71-0.75)[e]
Suburban	1.08 (1.05-1.11)[e]	0.90 (0.87-0.93)[e]	0.73 (0.71-0.75)[e]
Rural	1.01 (0.96-1.06)	0.86 (0.82-0.90)[e]	0.72 (0.70-0.75)[e]
Small town	0.90 (0.83-0.98)[e]	0.80 (0.76-0.84)[e]	0.71 (0.68-0.73)[e]
p-value for interaction[c]	0.02[e]	<.001[e]	0.27

Abbreviation: HDU=high density urban

Cause-specific hazards models adjusted for year of dialysis initiation, age, race and ethnicity, sex, cause of ESKD, employment status, body mass index (BMI), nephrology care, comorbidities (cancer, hypertension, diabetes, peripheral vascular disease, atherosclerotic heart disease, congestive heart failure, chronic obstructive pulmonary disease, drug use, alcohol use, tobacco use), and functional status.

[a]Neighborhood disadvantage score: nine domains (built environment disadvantage, criminal injustice, education disadvantage, unemployment, housing instability, poverty, social fragmentation, transportation barrier, and wealth inequality). As increasing values of the nine domains indicated more disadvantages, the naming convention was revised to reflect the appropriate interpretation of the domains and to improve clarity. https://www.sreindex.com/

[b]Race and ethnicity: non-Hispanic White, non-Hispanic Black, Hispanic and non-Hispanic Asian (Asian American, Native Hawaiian, and Pacific Islander).

[c]P-value for the interaction between neighborhood disadvantage score and race and ethnicity; neighborhood disadvantage score and US region; neighborhood disadvantage score and urbanicity

[d]Modified Rural-Urban Commuting Area [RUCA] Codes defined by US Department of Agriculture; high-density urban, suburban, rural, and small town. https://www.sciencedirect.com/science/article/pii/S2590291122000043

[e]Associations that are statistically significant (p<0.05)

© 2025 Li Y et al. *JAMA Network Open.*

eTable 9. [Sensitivity Analysis – National Tertiles] Residential neighborhood disadvantage and time to live-donor kidney transplantation (LDKT) and preemptive kidney transplantation (KT) among KT candidates (aged≥18), stratified by race and ethnicity, US region, and urbanicity (2015-2021; N=95,068)

	Adjusted Hazard Ratio (aHR) (95% Confidence Interval) Residential Neighborhood Disadvantage Score[a]					
	LDKT			Preemptive KT		
	**Low**	**Medium**	**High**	**Low**	**Medium**	**High**
**Overall**	Reference	0.82 (0.79-0.86)[e]	0.65 (0.62-0.68)[e]	Reference	0.75 (0.72-0.79)[e]	0.63 (0.59-0.67)[e]
**Race/Ethnicity**[b]						
Asian	0.43 (0.39-0.48)[e]	0.38 (0.32-0.44)[e]	0.26 (0.20-0.34)[e]	0.39 (0.34-0.44)[e]	0.30 (0.24-0.37)[e]	0.21 (0.14-0.30)[e]
Black	0.48 (0.44-0.53)[e]	0.34 (0.31-0.38)[e]	0.23 (0.21-0.25)[e]	0.30 (0.26-0.34)[e]	0.24 (0.21-0.28)[e]	0.23 (0.20-0.26)[e]
Hispanic	0.71 (0.65-0.78)[e]	0.55 (0.50-0.60)[e]	0.50 (0.46-0.54)[e]	0.51 (0.44-0.57)[e]	0.35 (0.30-0.40)[e]	0.27 (0.23-0.31)[e]
White	Reference	0.85 (0.81-0.90)[e]	0.69 (0.65-0.73)[e]	Reference	0.76 (0.72-0.80)[e]	0.63 (0.58-0.67)[e]
p-value for interaction[c]	<.001[e]	<.001[e]	<.001[e]	.001[e]	.001[e]	.001[e]
**US Region**						
Northeast	Reference	0.82 (0.75-0.90)[e]	0.59 (0.51-0.68)[e]	Reference	0.66 (0.59-0.74)[e]	0.58 (0.49-0.70)[e]
Midwest	1.06 (0.99-1.14)	0.96 (0.89-1.04)	0.80 (0.72-0.88)[e]	1.11 (1.03-1.20)[e]	0.89 (0.81-0.97)[e]	0.68 (0.60-0.77)[e]
South	1.18 (1.10-1.26)[e]	0.88 (0.82-0.94)[e]	0.69 (0.64-0.73)[e]	0.98 (0.90-1.07)[e]	0.74 (0.68-0.81)[e]	0.62 (0.57-0.68)[e]
West	0.79 (0.73-0.85)[e]	0.60 (0.55-0.66)[e]	0.42 (0.37-0.48)[e]	0.75 (0.69-0.82)[e]	0.57 (0.51-0.64)[e]	0.47 (0.40-0.55)[e]
p-value for interaction[c]	<.001[e]	<.001[e]	<.001[e]	<.001[e]	.001[e]	<.001[e]
**Urbanicity**[d]						
HDU	Reference	0.77 (0.72-0.83)[e]	0.59 (0.54-0.64)[e]	Reference	0.72 (0.66-0.79)[e]	0.58 (0.52-0.65)[e]
Suburban	1.04 (0.99-1.11)	0.82 (0.76-0.88)[e]	0.69 (0.64-0.75)[e]	1.10 (1.03-1.18)[e]	0.78 (0.72-0.85)[e]	0.68 (0.62-0.76)[e]
Rural	1.15 (1.05-1.26)[e]	1.02 (0.94-1.12)	0.71 (0.64-0.78)[e]	1.13 (1.02-1.25)[e]	0.78 (0.69-0.87)[e]	0.73 (0.65-0.82)[e]
Small town	0.91 (0.77-1.07)	0.95 (0.85-1.05)	0.74 (0.67-0.81)[e]	1.03 (0.87-1.22)	1.08 (0.96-1.20)	0.70 (0.62-0.80)[e]
p-value for interaction[c]	<.001[e]	<.001[e]	<.001[e]	0.07	<.001[e]	<.001[e]

Cause-specific hazards models adjusted for year of listing, age at listing, race and ethnicity, sex, cause of ESKD, body mass index (BMI), blood type, peak reactivity antibody, comorbidities (cancer, hypertension, diabetes, peripheral vascular disease, atherosclerotic heart disease, congestive heart failure, chronic obstructive pulmonary disease, drug use, alcohol use, tobacco use), and functional status.

[a]Neighborhood disadvantage score: nine domains (built environment disadvantage, criminal injustice, education disadvantage, unemployment, housing instability, poverty, social fragmentation, transportation barrier, and wealth inequality). As increasing values of the nine domains indicated more disadvantages, the naming convention was revised to reflect the appropriate interpretation of the domains and to improve clarity. https://www.sreindex.com/

[b]Race and ethnicity: non-Hispanic White, non-Hispanic Black, Hispanic and non-Hispanic Asian (Asian American, Native Hawaiian, and Pacific Islander).

[c]P-value for the interaction between neighborhood disadvantage score and race and ethnicity; neighborhood disadvantage score and US region; neighborhood disadvantage score and urbanicity

[d]Modified Rural-Urban Commuting Area [RUCA] Codes defined by US Department of Agriculture; high-density urban, suburban, rural, and small town. https://www.sciencedirect.com/science/article/pii/S2590291122000043

[e]Associations that are statistically significant ($p<0.05$)

© 2025 Li Y et al. *JAMA Network Open.*

eFigure 1. Residential neighborhood disadvantage and time to A) live-donor kidney transplantation (LDKT), and B) preemptive kidney transplantation (2015-2021). For ease of interpretation, we excluded the medium neighborhood disadvantage from the figure

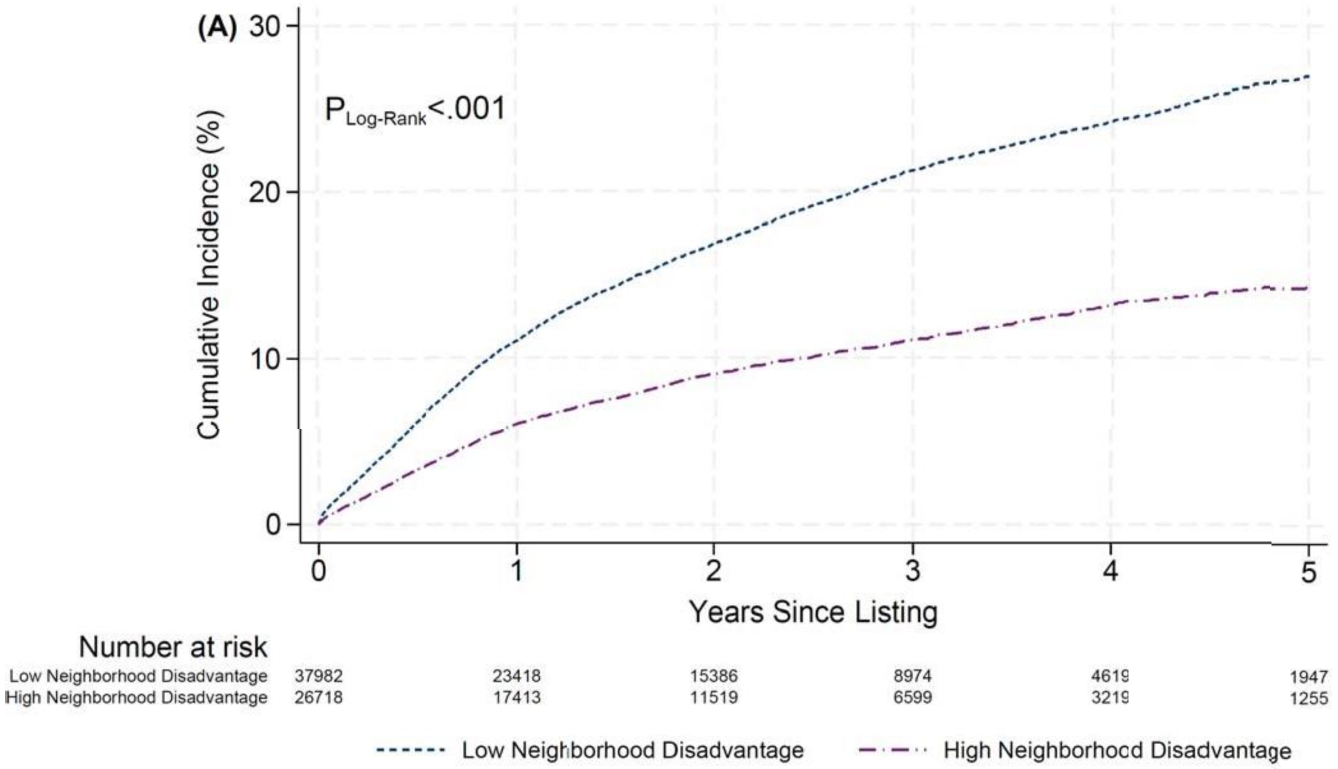

© 2025 Li Y et al. *JAMA Network Open.*

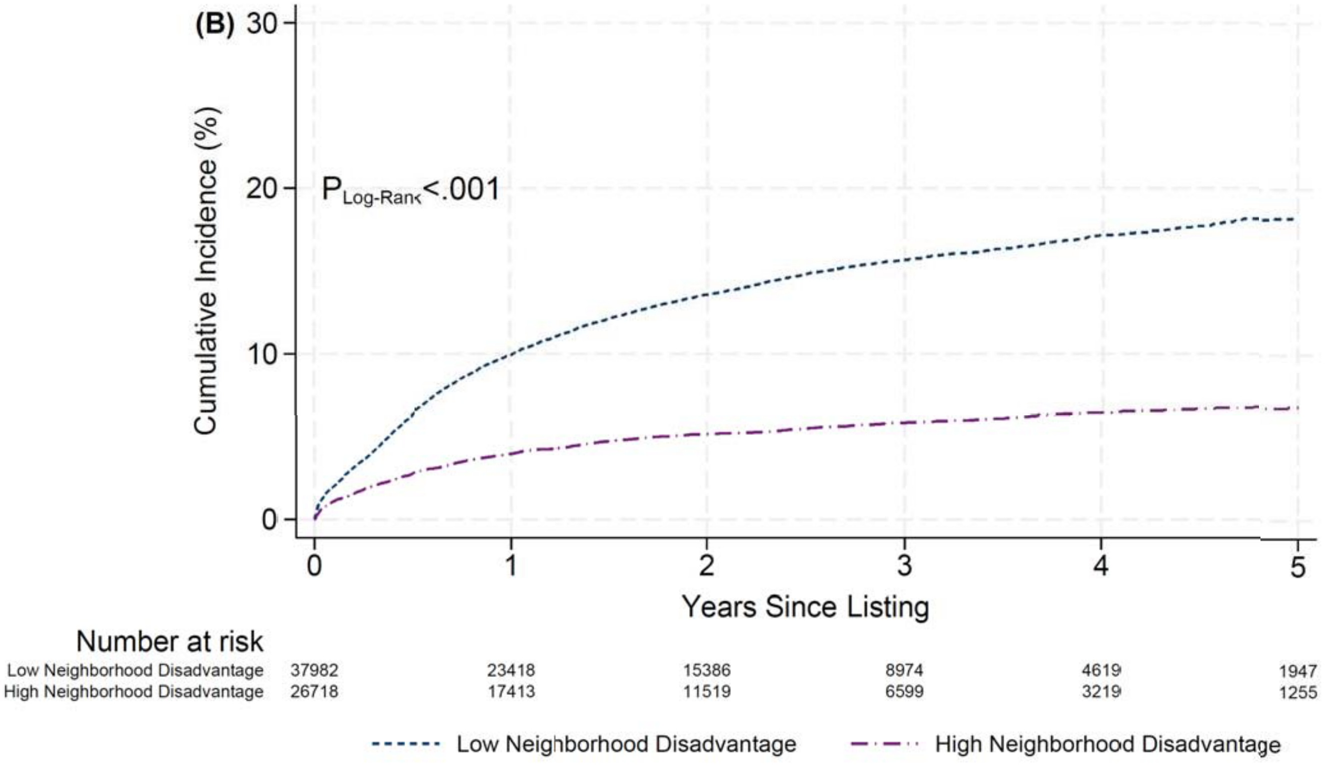

© 2025 Li Y et al. *JAMA Network Open.*
