# Supplementary material for: Residential Neighborhood Disadvantage and Access to Kidney Transplantation
Source: JAMA Netw Open. 2025 Dec 30;8(12):e2549679. doi: 10.1001/jamanetworkopen.2025.49679 (PMC12754678; doi:10.1001/jamanetworkopen.2025.49679)
Supplement: Supplement 2. — Data Sharing Statement [file jamanetwopen-e2549679-s002.pdf]

Data Sharing Statement

Li. Residential Neighborhood Disadvantage and Access to Kidney Transplantation. *JAMA Netw Open*. Published December 30, 2025. doi:10.1001/jamanetworkopen.2025.49679

Data
Data available: No

Additional Information
Explanation for why data not available: Data Availability Statement: The datasets used and/or analyzed during the current study are available from the United States Renal Data System (USRDS) upon Data Use Agreement (DUA) approval. Per the DUA between the authors and USRDS, the rerelease of the data or the deposition of data into publicly available repositories or to individuals is not allowed.
